# Immunodeficient NBSGW mouse strain allows chemotherapy modeling in AML patient‐derived xenografts

**DOI:** 10.1002/hem3.28

**Published:** 2024-01-27

**Authors:** Vilma Dembitz, Jozef Durko, Joana Campos, Sophie C. James, Hannah Lawson, Kamil R. Kranc, Paolo Gallipoli

**Affiliations:** ^1^ Centre for Haemato‐Oncology, Barts Cancer Institute Queen Mary University of London London UK; ^2^ The Institute of Cancer Research London UK; ^3^ Present address: Department of Physiology and Croatian Institute for Brain Research University of Zagreb School of Medicine Zagreb Croatia

Despite the recent introduction of many novel targeted therapies, the treatment backbone of acute myeloid leukemia (AML) in fit patients remains the combination of anthracycline (daunorubicin) and cytarabine (Ara‐C), also known as DA chemotherapy.^1^

Although in clinical trials most novel therapies are tested in combination with or against standard DA chemotherapy,^2^, ^3^ in AML preclinical models, novel inhibitors are often studied as single agents or compared to suboptimal control arms.^4^ It would be preferable for DA to be routinely incorporated in preclinical studies assessing new therapies to provide the best evidence for their clinical development.

AML patient‐derived xenografts (PDXs) are a valuable preclinical model to test the efficacy of novel drugs and combination regimens.^5^ PDXs typically retain the molecular and phenotypic characteristics of the primary disease^6^ and have been used to derive clinically applicable prognostic scores and therapeutic predictions.^7^, ^8^, ^9^ Generating PDXs requires immunocompromised recipient mice, which provide the permissive environment for adequate engraftment of AML leukemia stem cells and disease development.^10^ Most immunocompromised murine strains used in hematological research are homozygous for the severe combined immune deficiency mutation *Prkdc* ^*scid*^, commonly referred to as *scid*. The *scid* mutation causes a generalized defect in double‐strand DNA damage repair^11^ and leads to increased sensitivity to several DNA damaging agents. Specifically, *scid* mutant strains are more sensitive to the DNA‐damaging effects of ionizing radiation, and *scid*‐related toxicity is not limited to the hematopoietic compartment.^12^ Anthracyclines induce double‐strand DNA breaks^13^ and, given that the *scid* mutation causes defective double‐strand break repair,^11^ might lead to increased sensitivity of *scid* mutant mice to DA chemotherapy. Indeed, while administration of 3 mg/kg of doxorubicin and 100 mg/kg of Ara‐C chemotherapy, doses similar to clinical regimens in humans, is feasible in immunocompetent C57BL/6 mice,^14^ it is much more challenging in *scid* mouse strains. It is worth noting here that in murine studies DA refers to doxorubicin plus Ara‐C since doxorubicin is often substituted for the anthracycline daunorubicin, used in humans, due to the toxicity of daunorubicin in mice.^14^, ^15^ Previously Wunderlich et al. highlighted a maximum tolerated dose of 1.5 mg/kg of doxorubicin and 50 mg/kg of Ara‐C in several nonobese diabetic (NOD)‐*scid* mice strains.^15^ Moreover, given *scid* mice radiosensitivity,^12^ even this reduced dose could not be delivered when mice were previously exposed to sublethal irradiation, the conditioning protocol used for engraftment of most AML cells in NOD‐*scid* models.^15^ Although since the Wunderlich's report, other DA protocols containing reduced dose Ara‐C to 10 mg/kg while keeping doxorubicin at 1.5 mg/kg have been reported to be effective in NOD.Cg‐*Prkdc* ^scid^ *Il2rg* ^tm1Wjl^/SzJ (NSG) mice, they were still used in unconditioned recipient^16^ and it remains unclear if these reduced dose protocols can be delivered to preconditioned mice. Therefore, with the exception of the most aggressive samples able to engraft without preconditioning, DA chemotherapy cannot be tested at these doses in most AML PDXs generated using NSG recipients given the need for irradiation to achieve adequate engraftment.

Recently a new NOD‐*scid* strain produced by crossing the NSG strain with the C57BL/6J‐*Kit* ^W‐41J^/J (C57BL/6.*Kit* ^W41^) mice has been generated and is referred to as the NBSGW strain.^17^ It is permissive to human hematopoietic stem cell (HSC) engraftment without any irradiation, thus avoiding the hematopoietic, gastrointestinal, and neurological radiation toxicities.^17^ We observed that NBSGW mice are suitable recipients of human AML cell lines, which cause lethal disease and leukemic infiltration of hematopoietic organs. However, given that AML cell line‐derived xenografts (CDXs) are often highly proliferative, with a tendency to extra hematopoietic infiltration, the levels of hematopoietic organs' infiltration at terminal endpoints in NBSGW CDX are generally low, and comparable to that observed or reported in NSG recipients^18^, ^19^ (Figure 1A–C and Supporting Information S1: Figure 1A,B). Conversely, we struggled to engraft primary AML samples in NSG due to a significant proportion of animals succumbing to toxic radiation side effects at early time points preventing assessment of the engraftment beyond week 4 (Supporting Information S1: Figure 1C,D). This prompted us to switch to NBSGW as recipients for our PDXs and indeed we show that NBSGW mice can be grafted with primary AML samples, with successful myeloid engraftment in hematopoietic organs in all cases tested, regardless of the AML mononuclear cell source and genetic features even when transplanting a limited number of cells (Figure 1D,E and Supporting Information S1: Figure 1E). Thus, as previously reported for normal human HSC xenografts, NBSGW recipients might serve as better hosts for AML PDXs allowing the optimal use of often limited amounts of primary material without the requirement of potentially damaging preconditioning.

**Figure 1:**
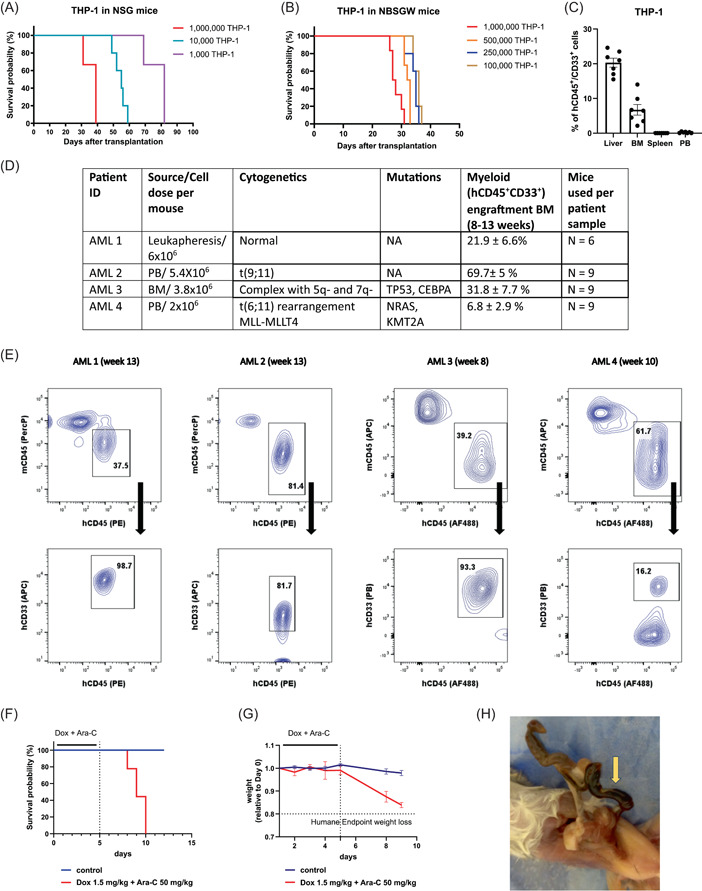
(A) Kaplan–Meier survival curve of NSG mice transplanted with increasing numbers of THP‐1 AML cell line (1000—1,000,000). (B) Kaplan–Meier survival curve of NBSGW mice transplanted with increasing numbers of THP‐1 AML cell line (100,000–1,000,000). (C) Engraftment at terminal endpoint of THP‐1 human myeloid blasts (hCD45^+^/hCD33^+^) in the liver, BM, and spleen and PB of transplanted NBSGW animals. Data are shown for each tissue as mean ± SEM. (D) Table showing average myeloid engraftment (percentage hCD45^+^/hCD33^+^ cells in total CD45^+^ cells) in the bone marrow of NBSGW mice transplanted with mononuclear cells of AML patients of different genetic backgrounds sampled from different sampling sources. Data are shown as mean ± SEM. (E) Representative flow cytometry plots showing engraftment of human hematopoietic cells (hCD45^+^) and human myeloid cells (hCD45^+^/hCD33^+^) in the bone marrow of NBSGW mice. (F) Kaplan–Meier survival curve of NBSGW mice treated with either Dox 1.5 mg/kg and Ara‐C 50 mg/kg (*n* = 9) or vehicle control (*n* = 6). (G) Changes in weight relative to Day 0 in NBSGW mice treated with either doxorubicin 1.5 mg/kg and Ara‐C 50 mg/kg or vehicle control. Data are shown at each time point as mean ± SEM. (H) Picture showing internal bleeding at necropsy in NBSGW mice treated with doxorubicin 1.5 mg/kg and Ara‐C 50 mg/kg. AML, acute myeloid leukemia; Ara‐C, anthracycline (daunorubicin) and cytarabine; BM, bone marrow; Dox, doxorubicin; NSG, NOD.Cg‐*Prkdc* ^scid^ *Il2rg* ^tm1Wjl^/SzJ; PB, peripheral blood.

Based on this, we considered it important to study the optimal chemotherapy delivery in this mouse strain. We first tested if NBSGW were able to tolerate 1.5 mg/kg of doxorubicin and 50 mg/kg of Ara‐C administered intravenously, a dose and delivery mode previously reported as being tolerated when given without irradiation in other NOD‐*scid* mice.^15^ Surprisingly, NBSGW mice significantly dropped their weight by more than 10% within 8 days from initiating DA chemotherapy and developed significant morbidity with evidence of internal bleeding. Indeed, by Day 10, all treated mice succumbed as a result of therapy‐related toxicity (Figure 1F–H). This is exactly the time when nadir in terms of blood counts would be expected after the end of this chemotherapy protocol in mice.^20^ Given these results and our experience in treating NBSGW mice with 50 mg/kg of Ara‐C as a single agent without any side effects,^21^ we reduced the dose of doxorubicin to either 0.5 or 1 mg/kg while maintaining Ara‐C dose at 50 mg/kg. At both these doses, mice weight was reduced but never more than 10% reaching a nadir at Day 10 with a quick recovery thereafter (Figure 2A). As expected, given the known effects of DA chemotherapy on normal bone marrow function, both doses of doxorubicin caused a significant transient decline in the white cell and platelet count while the drop in the hemoglobin was minor with full recovery of all blood counts by Day 21 post DA chemotherapy (Figure 2B–F). None of the treated mice died as a result of the DA chemotherapy at these doses. Based on these results, we therefore established 1 mg/kg of doxorubicin in combination with Ara‐C 50 mg/kg as a tolerable dose of DA in the NBSGW strain. Finally, we tested whether this DA dose still caused disease debulking and increased latency in AML xenografts models. We transplanted the aggressive human AML cell line MV4‐11 into NBSGW mice and introduced DA treatment 14 days after transplant. Reassuringly, we observed no lethality in response to chemotherapy, and even this reduced DA dose prolonged median survival by 11 days, consistent with previously published literature for MV411 xenotransplant models using higher DA doses in NSG recipients^22^ (Figure 2G). The efficacy of the reduced DA dose has also been validated by our group in independent experiments using murine *MLL* fusion‐driven leukemias transplanted into the NBSGW mice.^23^

**Figure 2:**
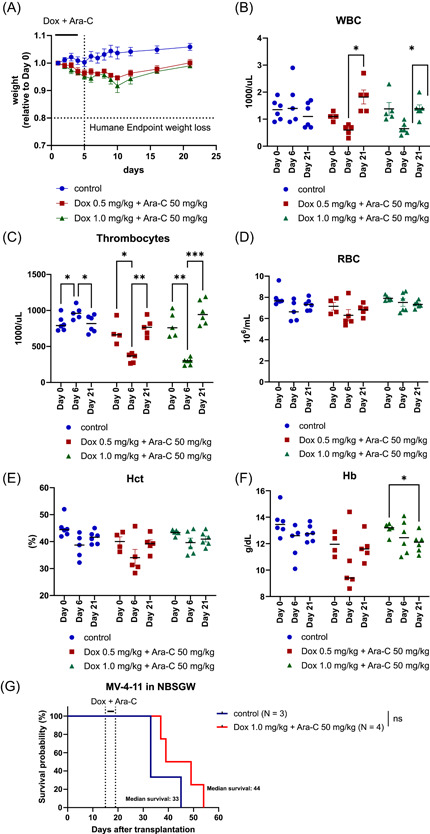
(A) Changes in weight relative to Day 0 in NBSGW mice treated with either doxorubicin 1.0 mg/kg and Ara‐C 50 mg/kg (*n* = 6), doxorubicin 0.5 mg/kg and Ara‐C 50 mg/kg (*n* = 5), or vehicle control (*n* = 6). Data are shown at each time point as mean ± SEM. (B–F) White blood cell (WBC), platelet, and red blood cell (RBC) count and hemoglobin (Hb) and hematocrit (Hct) levels at Day 0, 6, and 21 in NBSGW mice treated with either doxorubicin 1.0 mg/kg and Ara‐C 50 mg/kg, doxorubicin 0.5 mg/kg and Ara‐C 50 mg/kg, or vehicle control. Data are shown at each time point as a scatter dot plot for each individual mouse with the median highlighted. Statistical analysis was performed using two‐way ANOVA with Tukey's multiple comparisons test, *p < 0.05; **p < 0.01; ***p < 0.001. (G) Kaplan–Meier survival curve of NBSGW mice transplanted with MV‐4‐11 AML cell line and treated with either doxorubicin 1.0 mg/kg and Ara‐C 50 mg/kg (*n* = 4) or vehicle control (*n* = 3). Comparisons of survival curves were done using the log‐rank (Mantel–Cox) test. AML, acute myeloid leukemia; ANOVA, analysis of variance; Ara‐C, cytarabine; Dox, doxorubicin; ns, not significant likely due to small numbers; NSG, NOD.Cg‐*Prkdc* ^scid^ *Il2rg* ^tm1Wjl^/SzJ.

In conclusion, this is the first report, to our knowledge, of treating NBSGW mouse xenografts with DA chemotherapy. We show that the NBSGW mouse strain is even more sensitive than most NOD‐*scid* strains to DA‐induced toxicity requiring further reduction of the doxorubicin dose to 1 mg/kg. Reassuringly, we demonstrate that this dose still prolongs leukemic mouse survival and therefore using NBSGW strain for preclinical models of chemotherapy is feasible. Although chemotherapy modeling of AML PDXs can be successfully performed using NSG recipients as reported,^15^, ^16^ NBSGW appears to be more permissive to xenografting patient material. Compared to NSG, NBSGW would therefore allow testing more AML samples in PDX models of chemotherapy as no preconditioning irradiation is required to generate xenografts with this strain. The reasons behind the increased sensitivity of NBSGW to DA toxicity remain unknown, although we speculate it might derive from the combinatorial effects of the *scid* mutation with other polymorphism inherited via the crossing with the C57BL/6J‐*Kit* ^W‐41J^/J strain. It is worth noting that to overcome the inability to combine preconditioning with subsequent chemotherapy and the increased generalized sensitivity to genotoxic stress in NOD‐*scid* mice, alternative strains with *Rag1* ^*tm1Mom*^ mutation‐based immune deficiency have also been used.^24^ *Rag1* ^*tm1Mom*^ mutant mice tolerate higher doses of radiation while retaining the ability to engraft human HSC.^25^ This might be due to the fact that *Rag1* ^*tm1Mom*^ mutation only affects the differentiation and maturation of lymphocytes. However, NBSGW mice still provide an advantage compared to this latter strain as it allows for modeling AML and DA chemotherapy treatment across multiple AML samples in an organism not compromised by the toxic effects of irradiation on the bone marrow microenvironment and other organs. In conclusion, we show that NBSGW is permissive to AML chemotherapy modeling and identify its optimal dose for the benefit of the hematology research community. Given NBSGW permissiveness to PDXs, their adoption as hosts for modeling AML would allow the use, across most primary AML samples, of DA chemotherapy as a standard control arm and/or in combination with novel targeted therapies in preclinical studies. This, in turn, will increase the translational relevance of these studies.

## AUTHOR CONTRIBUTIONS

All authors designed the experiments. Vilma Dembitz, Jozef Durko, Joana Campos, Sophie C. James, and Hannah Lawson performed experiments. Vilma Dembitz, Kamil R. Kranc, and Paolo Gallipoli wrote and edited the manuscript. All authors reviewed and approved the manuscript.

## CONFLICT OF INTEREST STATEMENT

The authors declare no conflict of interest.

## FUNDING

This research was supported by the BCI Flow Cytometry Facility (CRUK Core Award C16420/A18066), Cancer Research UK (PG, Advanced Clinician Scientist fellowship, C57799/A27964), and Lady Tata Memorial Trust (VD, International Award for Research in Leukaemia).

## Supporting information

Supporting information.

## Data Availability

No big data were produced in this manuscript. Primary data used to generate figures are available upon request to the corresponding author.
