# Supplementary material for: Immunodeficient NBSGW mouse strain allows chemotherapy modeling in AML patient‐derived xenografts
Source: Hemasphere. 2024 Jan 27;8(1):e28. doi: 10.1002/hem3.28 (PMC10878184; doi:10.1002/hem3.28)
Supplement: Supplementary file 1 — Supporting information. [file HEM3-8-e28-s001.pdf]

SUPPLEMENTAL DIGITAL CONTENT (SDC)

Supplemental Materials and Methods

All leukemic cell transplants in this study were performed by intravenous injection of leukemic cells resuspended in 200 µL PBS in the tail vein of 8-12 weeks old immunocompromised mice. Animals were housed in specific pathogen free conditions in individually ventilated cages and received sterile drinking water and irradiated food.

Chemotherapy was delivered in a 5 day protocol in which animals intravenously received corresponding doses of doxorubicin and cytarabine mixed in the same syringe for 3 days combined with intravenous administration of 50 mg/kg cytarabine for two additional days.

Transplantation of human hematopoietic cells in NSG mice

For establishment of cell-line derived xenograft (CDX), human AML cell line THP-1 was transplanted in NSG mice in increasing cell numbers (1 000 – 1 000 000). Animals were culled upon reaching humanized endpoints and survival analysis was performed.

For establishment of patient derived xenograft (PDX), two primary AML samples were T-cell depleted and transplanted into previously sub-lethally irradiated (2.25 Gy) NSG mice ($5x10^6$ cells per mouse). Animals were culled upon reaching humanised endpoints due to pronounced irradiation toxicity and surviving mice were culled at 4 weeks from transplantation to determine potential human hematopoietic ($hCD45^+$) engraftment.

Transplantation of human hematopoietic cells in NBSGW mice

For establishment of cell derived xenografts, human AML cell line THP-1 was transplanted in NBSGW strain in increasing cell numbers (100 000 – 1 000 000) and MV-4-11 cell line was transplanted in concentration 500 000 cells per mouse. Animals were culled upon reaching humanized endpoints and human hematopoietic cells ($hCD45^+$) engraftment was assessed in the bone marrow, peripheral blood, spleen and liver of transplanted animals. THP-1 model was compared with the one established in NSG strain, and MV-4-11 model with the data in NSG strain previously published in literature.[1]

For establishment of patient derived xenograft (PDX), primary AML samples were T-cell depleted and transplanted into NBSGW mice in concentration described in Figure 1D. Human hematopoietic cells ($hCD45^+$) engraftment was assessed in the bone marrow of transplanted animals either by post mortem collection (samples AML1 and AML2) or by bone marrow sampling from live animals (samples AML3 and AML4) 8 to 13 weeks after transplant. As it is described that NBSGW strain is highly permissive for engraftment of B-cell lineage,[2,3] we determined the ratio of B-cells ($hCD45^+/hCD33^-/hCD19^+$) and myeloid cells ($hCD45^+/hCD33^+/hCD19^-$) for the sample (AML4) where the majority of $hCD45^+$ cells in the engrafted population did not correspond to myeloid cells. As a negative control, we used a sample where total engrafted population was comprised of myeloid cells (AML3). This analysis confirmed previous reports that high levels of B-cell engraftment does not impede myeloid engraftment in NBSGW strain nor AML manifestation. However it also stresses the importance of specifically checking myeloid engraftment when working with AML PDX particularly when using highly permissive recipients as in this case.

Supplemental references

1. Saland E, Boutzen H, Castellano R, et al. A robust and rapid xenograft model to assess efficacy of chemotherapeutic agents for human acute myeloid leukemia. *Blood Cancer Journal*. 2015/03/01 2015;5(3):e297-e297. doi:10.1038/bcj.2015.19

2. Chen D-W, Huang T, Huang Y, et al. Developing a Humanized Murine Model for Co-Transplantation of Acute Myeloid Leukemia. *Blood*. 2022;140(Supplement 1):5776-5777. doi:10.1182/blood-2022-158615

3. McIntosh Brian E, Brown Matthew E, Duffin Bret M, et al. Nonirradiated NOD,B6.SCID Il2rγ$^{-/-}$ Kit$^{W41/W41}$ (NBSGW) Mice Support Multilineage Engraftment of Human Hematopoietic Cells. *Stem Cell Reports*. 2015;4(2):171-180. doi:10.1016/j.stemcr.2014.12.005

Supplemental Figure 1

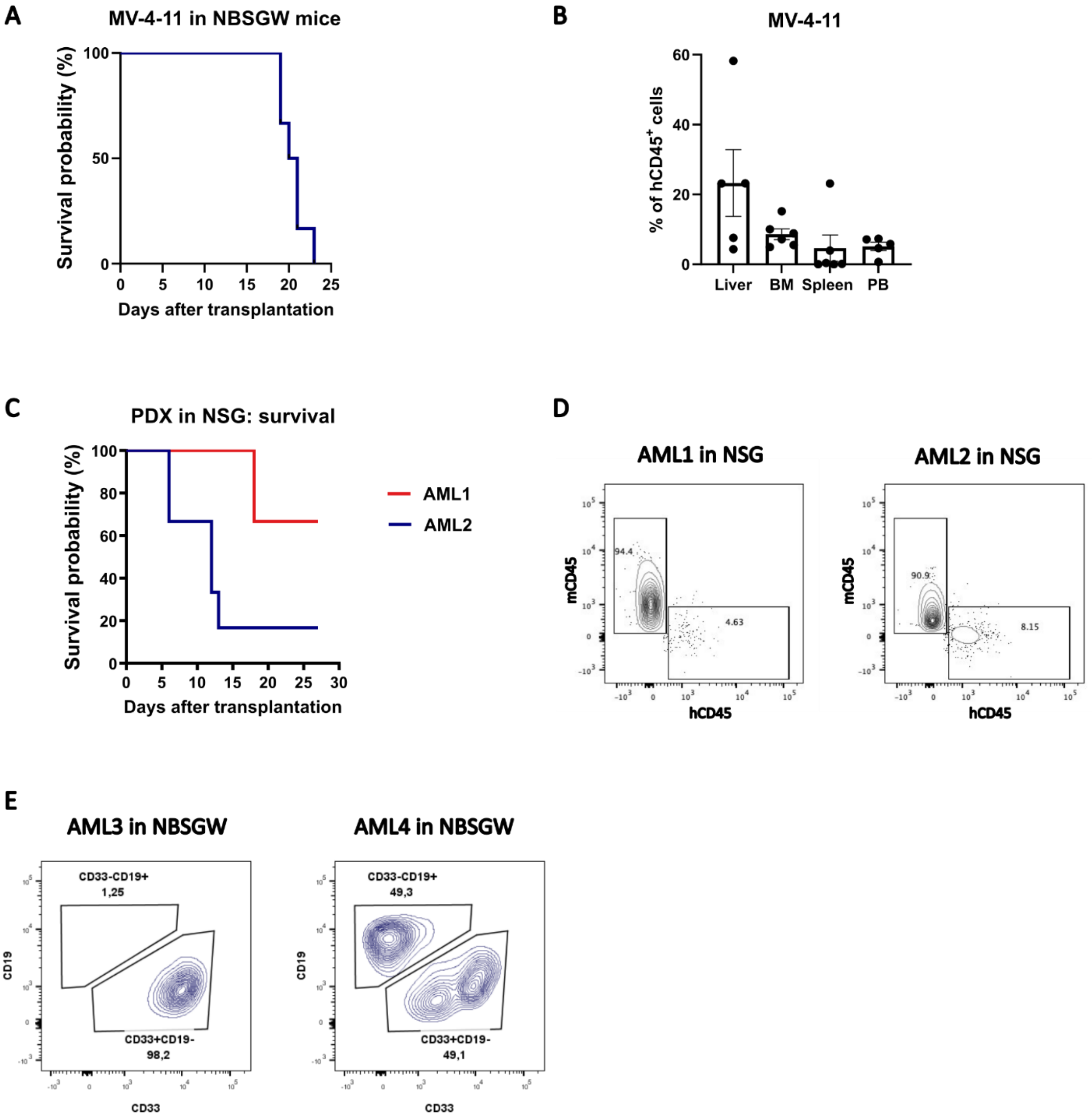

Supplemental Figure 1. A) Kaplan-Meier survival curve of NBSGW mice transplanted with MV-4-11 AML cell line (500 000 cells per mouse). B) Engraftment of human hematopoietic cells (hCD45+) in the liver, bone marrow (BM), spleen and peripheral blood (PB) of transplanted NBSGW animals. Data is mean ± SEM. C) Kaplan-Meier survival curve of irradiated NSG mice (2.25 Gy) transplanted with primary AML samples. Animals culled for engraftment analysis at 4 weeks from transplant were excluded from survival analysis. D) Representative flow cytometry plots of human hematopoietic cells ($hCD45^{+}$) engraftment in the bone marrow of transplanted NSG mice at 4 weeks after transplant. E) Representative flow cytometry plots showing ratio of myeloid cells ($CD33^{+}$) and B-cells ($CD19^{+}$) in the population of human hematopoietic cells ($hCD45^{+}$) in the bone marrow of NBSGW mice transplanted with primary AML samples.
